# Resource factors for mental health resilience in early childhood: An analysis with multiple methodologies

**DOI:** 10.1186/1753-2000-7-6

**Published:** 2013-02-22

**Authors:** Lauren R Miller-Lewis, Amelia K Searle, Michael G Sawyer, Peter A Baghurst, Darren Hedley

**Affiliations:** 1 Discipline of Paediatrics, School of Paediatrics and Reproductive Health, University of Adelaide, Adelaide, South Australia 5005, Australia; 2 Research and Evaluation Unit, Women’s and Children’s Health Network, 72 King William Road, North Adelaide, South Australia 5006, Australia; 3 Centre for Traumatic Stress Studies, School of Population Health, University of Adelaide, Adelaide, South Australia, 5005, Australia; 4 Public Health Research Unit, Women’s and Children’s Health Network, 72 King William Road, North Adelaide, South Australia, 5006, Australia; 5 School of Population Health, University of Adelaide, Adelaide, South Australia, 5005, Australia; 6 Child Development Center, Nationwide Children’s Hospital and Ohio State University, Columbus, Ohio, USA

**Keywords:** Resilience, Early childhood, Family adversity, Mental health, Child-adult relationships, Self

## Abstract

**Background:**

Given that relatively little is known about the development of resilience in early childhood, this longitudinal study aimed to identify preschool resource factors associated with young children’s mental health resilience to family adversity.

**Methods:**

A community sample of 474 young Australian children was assessed in preschool (mean age 4.59 years, 49% male), and again two years later after their transition into formal schooling. At each assessment, standard questionnaires were used to obtain ratings from both parents and teachers about the quality of children’s relationships with parents and teachers, children’s self-concept and self-control, mental health (Strengths and Difficulties Questionnaire), and family adversities (including stressful life events and socioeconomic disadvantage).

**Results:**

Greater exposure to cumulative family adversities was associated with both greater teacher- and parent-reported child mental health difficulties two years later. Multiple methodologies for operationalizing resilience were used to identify resources associated with resilient mental health outcomes. Higher quality child–parent and child-teacher relationships, and greater child self-concept and self-control were associated with resilient mental health outcomes. With the exception of child-teacher relationships, these resources were also prospective antecedents of subsequent resilient mental health outcomes in children with no pre-existing mental health difficulties. Child–parent relationships and child self-concept generally had promotive effects, being equally beneficial for children facing both low- and high-adversity. Child self-control demonstrated a small protective effect on teacher-reported outcomes, with greater self-control conferring greater protection to children under conditions of high-adversity.

**Conclusions:**

Findings suggest that early intervention and prevention strategies that focus on fostering child-adult relationship quality, self-concept, and self-control in young children may help build children’s mental health and their resilience to family adversities.

## 

Significant mental health difficulties such as depressive-, hyperactive- and conduct-disordered symptomatology are experienced by about one in eight children [1,2]. These problems tend to persist and are associated with adverse psychosocial, educational, and health outcomes in adolescence and adulthood (e.g., [1,3]). Consequently, early childhood is considered an opportune time for implementing early intervention strategies aimed at altering the trajectory of pathways that lead to the emergence of these mental health difficulties [4,5]. Research indicates it is more effective and economical to intervene early to promote optimal development, as opposed to intervening after problems become established (e.g., [4,6]).

It is well documented that numerous types of family adversity (e.g., socio-economic disadvantage, adolescent parenthood, parental separation, parental mental health problems, stressful family life events) increase the likelihood that children will develop mental health difficulties (e.g., [2,7-12]). Moreover, such adversities tend to co-occur, and their cumulative effects are associated with the development of childhood mental health difficulties, with evidence suggesting that it is the number rather than a specific type of an individual adversity in isolation that has the greatest impact [11,13-15].

However, there is great individual variation in children’s response to adversity, and many children exposed to adversity escape relatively unscathed and instead function adequately [13,16]. Resilience refers to this process of positive adaptation despite exposure to significant adversity [17,18]. Adversity is considered ‘significant’ when it is commonly associated with poorer outcomes, and Adaptation is ‘positive’ if functioning in a developmentally appropriate domain (e.g., mental health) is “better than expected” given the level of adversity experienced [17,19]. Because resilience is a phenomenon that can only be considered within the context of adversity, it is not a fixed or immutable trait that a person ‘has’ – a person may exhibit resilient outcomes in one context or domain but not in another [20]. Examining resilient outcomes adds to our knowledge because it involves investigating functioning (or ‘competence’) that is ‘unexpected’, due to the presence of adversity. By studying resilient outcomes in children, it is possible to identify resource factors that enable children to adapt positively to adversity. This is important because adversities are often deep-seated family and social problems that are difficult to change. A better understanding about why some children are more resilient than others within the context of adversity has the potential to guide the development of new evidence-based early interventions designed to better prepare children to cope with current and future adversity [4,18]. Understanding factors that promote resilient outcomes in at-risk children faced with adversity helps to ensure that children with the odds stacked against them will benefit from prevention programs by targeting resources known to protect at-risk children from poor developmental outcomes [17].

Given that relatively little is known about the development of resilient outcomes in early childhood [21], this longitudinal study aimed to identify characteristics of 4 year-old preschool children, their families, and their preschools which predicted ‘better-than-expected’ (i.e., resilient) outcomes on mental health difficulties two years later within the context of cumulative family adversity.

## Resource factors for mental health resilience

Specific resource factors or assets may have the potential to buffer or ameliorate the detrimental effects of adversity and lead to resilient outcomes. A considerable body of research has identified a core set of resources that are associated with resilience across various adversities and developmental outcomes. These are grouped in three domains: (a) children’s internal characteristics and strengths, e.g., self-esteem, self-efficacy, self-control; (b) family characteristics and relationships, e.g., child–parent closeness, parenting styles; and (c) characteristics of children’s social (particularly school) environment, e.g., student-teacher relationships, school-quality [17,22-24].

Internal child characteristics such as self-concept, including self-esteem and self-efficacy, have mostly been associated with resilience in older children and adolescents. In longitudinal studies, Werner and Smith [25,26], Masten and colleagues [12], and Elder and Conger [27] found that positive self-worth (self-esteem and self-efficacy) was longitudinally predictive of psychosocially resilient outcomes in adolescents within the context of family adversity and stress. In the Rochester child resilience project [28,29], self-esteem and perceived self-competence were associated with resilient adjustment for school-aged children experiencing stressful life events, but this was not the case in a similar study conducted in Australia [30]. However support for a relationship between positive child self-concept and psychosocial resilience is also provided by other studies involving at-risk children and adolescents exposed to specific adversities such as socio-economic disadvantage [31-33], family disintegration [31,34,35], and maternal depression [36].

Children’s self-control or emotional regulation may also buffer adversity and promote adaptive outcomes by enabling children to respond positively to stressful circumstances [37,38]. In two cross-sectional studies of socio-economically deprived preschool children attending Head Start, greater emotional regulation was associated with fewer internalising problems [39], fewer conduct problems and more pro-social behaviour [40]. Longitudinal studies of at-risk young children growing up in poverty have found that toddler emotional/behavioural regulation and attentiveness/persistence on tasks is predictive of fewer behavioural problems 3 to 4 years later [41,42]. Emotional regulation (including lower negative emotionality and greater inhibitory control) has demonstrated both concurrent and longitudinal associations with adaptive mental health outcomes in other high-adversity samples, including children exposed to domestic violence, maternal depression, impoverished minority youth, children experiencing maltreatment and cumulative family adversities, and homeless children [37,43-51]. Further studies with school-aged children found that self-regulation moderated the association between socio-contextual family adversities and mental health outcomes, signifying the potential role of self-regulation as a protective factor in the context of family adversity [43,52].

Supportive child–parent relationships characterised by warmth and closeness have been found to consistently predict mental health resilience in children. For example, in children from the Kauai longitudinal study exposed to cumulative family adversities, the resilient youth had more supportive relationships and interactions with capable parents than non-resilient youth [25,26]. The association between the quality of child–parent relationships and positive mental health child outcomes is demonstrated in several other longitudinal studies within different adversity contexts including socio-economic disadvantage [27,33,42,53-59], parental death and divorce [35,60,61], stressful life events [12,62], and child maltreatment [31,33,63] some of which focussed on early childhood outcomes [42,53-55,57-59,63]. In longitudinal studies of young children examining family relationships as a moderator of the association between adversity exposure and child mental health symptoms, O’Grady and Metz [64] found that greater family support provided by parents for children buffered the effect of stressful life event exposure on children’s emotional and behavioural problems, Malmberg and Flouri [65] found that mother-child relationship quality buffered the effect of socio-economic disadvantage on children’s emotional symptoms, and Maughan and colleagues [44] found that maternal negative parenting moderated the effect of maternal depression on young children’s perceptions of social acceptance. In direct contrast, Calkins and colleagues [66] found that a more responsive relationship between parent and toddler was associated with more externalising and internalising behaviour in 5 year old children exposed to high family adversity.

There is also a small amount of evidence that close supportive relationships with teachers are associated with resilience in children faced with adversity. For example, the resilient adolescents from the Kauai longitudinal study frequently had a favourite teacher who became a role model for them [25,26]. In a longitudinal study of children aged 4 to 8 years, Peisner-Feinberg and colleagues [67] found teacher-child closeness was more strongly related to lower levels of behaviour problems among children identified at-risk due to low maternal education, compared with their low-risk peers. Similar findings have been obtained from cross-sectional studies of preschool children at-risk due to socio-economic deprivation [40,68]. In Qualitative studies of Australian and South-African children experiencing adversity, those identified as ‘resilient’ by their teachers frequently made positive comments about special caring teachers who had a positive impact on their wellbeing [69,70].

In summary, considerable evidence exists for the role of each of the groups of child, family, and social/school factors in the development of mental health resilience. However, some limitations deserve mention. First, the majority of this research has focussed on resilience in middle childhood and adolescence [21]. In comparison, few studies have investigated resource factors during preschool, or resilient outcomes in young children across the preschool to school transition, which is considered a critical period of rapid developmental change [59]. Thus, it is unclear if promoting these factors during preschool will improve mental health outcomes in young children exposed to family adversity. Second, it is notable that the school environment, and particularly the potential role of teachers, has received far less empirical attention than other resource domains. As a result, few studies have examined resource factors from all three child, family, and social/school domains in the same study (notable exceptions include [25,26] and [42]). Without knowing what their unique contributions are, it is unclear whether one resource may be more important than another. This is an important omission, given that evidence already exists of the influence of resources from all three domains. Third, a considerable proportion of studies examine single adversity factors in isolation (e.g., maltreatment, poverty). Comparatively fewer studies [15,25-27,66,71] have examined cumulative family adversities including combinations of socio-economic factors, stressful life events, parental mental health, and parental separation. This is considered problematic because “focusing on a single risk factor does not address the reality of most children’s lives” (p.367) [71].

Finally, the vast majority of research on child resilience has been conducted in the US and UK. Conducting research in other countries such as Australia is important because resource factors relevant to resilience may be context and culture specific [72-74]. It is not known whether Australian children may demonstrate unique developmental patterns and responses to adversity. While these countries are all English-speaking multicultural western societies, the different distributions of socio-economic disadvantage, greater income mobility, less spatial concentration of public housing, and the nationwide universal provision of free preschool for all 4–5 year old children in Australia make it difficult to know how directly applicable findings from the US and UK would be to Australian children [72,75]. Only a handful of studies have investigated mental health resilience in Australian children (e.g., [30,36,51,69,74-79]), with the evidence for young Australian children limited to studies finding support for positive child–parent relationships and home environments as correlates of mental health in the context of family disadvantage and stress [51,76,78,79]. There is much more knowledge to be gained in this context.

### Multiple methodologies for measuring resilience

Resilience is a concept that is inferred on the basis of associations between the levels of (a) exposure to adversity and (b) positive adaptation or positive adjustment outcomes, and therefore it cannot by directly measured [18,24,80]. There is no ‘gold standard’ for operationalising the concept of resilience, and several different approaches are currently used to combine adversity and adjustment levels to measure resilient outcomes. When this occurs it can be difficult to compare results from different studies of resilience as it is possible they may not actually be measuring the same concept or phenomenon [24,80,81].

Broadly, methods of measuring resilience can be classed as variable-centred or person-centred approaches. Variable-centred approaches examine statistical associations between measures of adversity, hypothesised resource factors, and developmentally-relevant functioning, using regression-based analyses. If a factor modifies (i.e., reduces) the negative effects of adversity on functioning, then it is labelled ‘protective’, and it is implicated in resilience among the children for whom the risk and protective factors co-occur [82,83]. Researchers typically test such modifying effects using a statistical interaction term between the adversity and hypothesised protective variables. The ‘statistical interaction’ approach draws on the statistical power of the whole sample. However, the children who meet the criteria for resilience are never explicitly identified, and thus which children are deemed resilient remains unknown [84]. Additionally, statistical interaction terms within regression can lack adequate statistical power to fully and reliably detect real interactions, leading some researchers to caution against relying on statistical interaction terms [16,82,84].

Two other variable-centred approaches, used in combination, can address these two main limitations. First, the ‘residuals’ approach can identify resilient children who, in a statistical sense, are ‘doing better than expected’, while also keeping all data as continuous. With this approach, when regressing adjustment on adversity, the difference between a child’s actual adjustment score and his/her adjustment score predicted by adversity (i.e., the standardised residual scores) can be utilised as a continuous vulnerability-to-resilience score. Children with positive residual scores (i.e., falling above the regression line fitted) show ‘better than expected’ adaptation than predicted by their exposure to adversity, and are considered resilient (with the size of the residual indicating their level of resilience). This residuals methodology is a relatively innovative approach [17] and variants of it have been used in several resilience studies [27,85-88]. Second, the ‘residuals’ approach can be used with a ‘multiple-groups’ approach, where main-effects regression analyses predicting resilience residual scores are run separately for low- and high-adversity groups [89-92]. Subsequent effect sizes for each group can then be compared to examine the specificity of processes (i.e., whether a resource is a general ‘promotive factor’ associated with good outcomes in both low- and high-adversity children, or a specific ‘protective factor’ with unique benefits only for high-adversity children) while avoiding the statistical problems related to statistical interaction terms [17].

In contrast to variable-centred approaches, person-centred approaches involve identifying a group of resilient children (who experience high adversity but exhibit adequate adjustment), and comparing their characteristics with other groups of children showing different patterns of adversity and adjustment, in order to identify resource factors associated with resilience (e.g., [12,25,90]). Using Masten and colleagues [12] taxonomy as an example, if four groups of children with divergent outcomes are identified - two high-adversity groups identified as either ‘resilient’ (good adjustment) or ‘maladaptive’ (poor adjustment), and two low-adversity groups classified as ‘competent’ (good adjustment) or ‘highly vulnerable’ (poor adjustment) - it is possible to determine if a resource is truly protective rather than generally promotive by examining if resource levels differ between ‘resilient’ and ‘maladaptive’ children, but not between ‘competent’ and ‘highly vulnerable’ children. A key advantage of the person-centred approach is that it better reflects resilience as it actually occurs naturally within the whole child, rather than through associations between variables. Due to this, manifestly resilient children can actually be identified [17,18,23,36]. However, reducing the vast individual differences present in early childhood development into broad dichotomous categories may be problematic, as valuable detail becomes lost, particularly if the sample size is substantially reduced by selecting more extreme subgroups only [84,93]. Furthermore, if cut-points are somewhat arbitrarily defined (particularly a median-split) without a solid reason to suspect different effects between the groups created, then effects that occur within rather than between groups may be obscured [84].

Despite the considerable methodological variation in resilience studies, the fact that a common set of child, family, and social resources have been consistently recognised in resilience suggests that these resources are all implicated in the same underlying phenomenon, and support the validity of resilience as a construct [18,23,80]. Given their seemingly universal importance, these particular resource factors could be quite useful for further systematic exploration of the resilience construct, and critical examination of its measurement. However, researchers have rarely addressed whether similar variables emerge as significant resources while employing multiple resilience methodologies within the same sample. Inferences have needed to be made across studies, when many other factors could not be accounted for, such as sample characteristics. Given the relative strengths and weaknesses of both variable- and person-focussed resilience methodologies, it seems sensible to use both types of methods in combination in the same study (e.g., [12,84]).

As different resilience measurement approaches are rarely used in a single study, little information exists regarding how different methodologies may affect results (whilst holding constant the sample and variable measures). Masten and colleagues [12] conducted both variable-centred analyses (examining whether resource variables buffered the negative impact of adversity using regression interactions), and person-centred analyses (examining whether the same resource factors distinguished between ‘Resilient’, ‘Maladaptive’ and ‘Competent’ groups of children in MANOVAs). However, the fourth ‘Highly Vulnerable’ group (low adversity + poor adjustment) was omitted because it was an ‘empty cell’, so the possibility that associations between resources and positive adjustment differed between high-adversity and low-adversity children could not be examined. Thus, although complementary, their variable- and person-centred approaches were not directly comparable (see also [46,49,94-96]). To our knowledge, only one study has assessed interactive effects within both variable- and person-centred analyses. Lengua [43] examined whether resource levels discriminated not only between two high-adversity groups (e.g., ‘resilient’ vs. ‘maladaptive’), but also between two low-adversity groups (e.g., ‘competent’ vs. ‘highly vulnerable’), using logistic regressions. Findings were then compared with those from linear regression interaction terms. However, these methodologies were not fully comparable because they used a different adjustment variable – the adjustment variables were examined separately within variable-centred analyses, but were combined into a composite adjustment variable for person-centred analyses.

### The present study

The aim of the present study was to investigate child, family, and preschool resource factors associated with the development of resilient mental health outcomes during the early childhood years. We hypothesised that (a) children’s characteristics (higher self-esteem, self-efficacy, and self-control), (b) better quality child–parent relationships, and (c) better quality child-teacher relationships during preschool, would be associated with greater mental health resilience in children two years later once at school. To achieve this aim, we utilised the four different methodological approaches for operationally defining resilient outcomes (as described above). This strategy allowed the investigation of whether similar resource factors emerged as predictive of resilient outcomes in young children when different methodological techniques were used. To our knowledge, this is the first study to analyse results from directly comparable techniques for operationally defining resilient outcomes.

There are several unique aspects to this study. We add to the relatively small body of literature on resilient outcomes in young children, and to the limited information regarding the various potential resources in the child, family, and school domains that children experience during the preschool year [21]. This may inform early intervention efforts designed to maximise positive development in young children and intervene before mental health difficulties become entrenched [4,5]. The present study also builds upon previous research by longitudinally investigating resource factors associated with resilient outcomes in a contemporary cohort of young children. Finally, the present study represents one of the first investigations of mental health resilience in the context of cumulative family adversities in Australian children. These aspects are important given that resilience is considered a contextually and culturally embedded phenomenon, and a multiply-determined and mutable developmental process [20,74].

## Method

### Participants

Participants were the families of 485 children attending the 27 government-funded preschools in one South Australian government schooling district (at Time 1, mean age = 4.59 years, *SD* = 0.33, age range = 3 to 5 years, 49% male). This district is quite diverse, encompassing suburban, rural and remote areas, with some of these ranked at the highest levels of socio-economic disadvantage in Australia. The demographic characteristics of this district overall resemble those for South Australia as a whole [97].

In 2006, participation was sought from all families of children attending preschool ^a^ within the district. At baseline, both a parent survey and a teacher survey were completed for 601 children (representing 62% of all district preschoolers). Based on school district records, the 62% of children recruited were of similar age and gender distribution to the preschool children in the whole district, but the percentage of children of Aboriginal/Torres Strait Islander (ATSI) descent was somewhat lower in the participating sample than in the school district population (1.4% versus 3.9%). This suggests the study findings may not be as generaliziable to ATSI children. Children were assessed two years later after they had commenced formal schooling. Both parent and teacher surveys were completed for 485 of these children (retention rate = 81%). At both assessments, the parent-reported surveys were completed by mothers for the majority of the sample (92% at both assessments). Eleven children were missing data for at least one of the study variables, so analyses were conducted using the remaining 474 children will full data. Table 1 provides demographic information about these 474 participating children.

**Table 1:** Child and family demographic background (n = 474)

***Time 1 variables***	***M (SD) or %***
Child age	4.59 (0.33)
Child Gender Female	51%
Family receives means-tested government pension/benefit	40.3%
Mother was adolescent (<21) when child born	7.2%
Father was adolescent (<21) when child born	2.0%
Child currently lives in a single parent family	12.9%
Child has 3 or more siblings living in home	8.9%
Number of stressful family life events in the last year	0.90 (1.17)
Parental psychological distress (GHQ) score	1.41 (2.49)
Above GHQ clinical cut-off	39.1%
Number of years child lived separate from one (or both) natural parents ^a^	0.50 (1.28)
Mother’s education level ^b^	2.51 (1.07)
Completed university qualifications	20.3%
Technical, trade, or further education (TAFE) certificate	33.4%
Completed high school Year 12 or equivalent	21.6%
Completed some years of high school or less	24.7%
Father’s education level ^b^	2.65 (1.03)
Completed university qualifications	11.2%
Technical, trade, or TAFE certificate or some university	42.7%
Completed high school Year 12 or equivalent	15.9%
Completed some years of high school or less	30.3%
Family paid employment level ^b^	1.74 (0.99)
Two parents employed full-time	6.3%
One parent full-time and one part-time	38.0%
One parent full-time or 2 parents part-time	39.9%
One parent part-time	7.2%
Both parents unemployed or an unemployed single parent	8.6%

*Note.*^a.^ i.e., Duration of parental separation ^b.^ Higher scores on these variables indicate less education and employment, respectively.

The children lost from the sample between assessments (*n=* 116) were significantly (i.e., *p* < .05) more likely to be living in a single parent family (28.7% vs. 13.6%) that was receiving a means-tested government pension/benefit (60.9% vs. 40.9%), had experienced more stressful life events in the past 12 months (1.4 vs. 0.9), had mothers who had not completed high school (37.8% vs. 25.1%) and fathers that were unemployed (24.1% vs. 10.4%), a large number of siblings (20.7% vs. 8.7%), and younger fathers (30.8 years vs. 32.4 years). Those children lost to attrition also had significantly greater levels of parent-reported (9.97 vs. 8.47) and teacher-reported (6.66 vs. 5.28) mental health difficulties at the initial assessment. Hence, those lost from the sample tended to be families with greater exposure to family adversities and children with greater mental health difficulties.

### Measures

Children’s primary care-giving parent and their current teacher completed the following standardised questionnaires at the baseline and follow-up assessments. The internal consistencies of the continuous-measured scale variables used in the present study were adequate, with Cronbach’s alphas ranging from .78 to .95 (see Table 2).

**Table 2:** Bivariate correlations between continuous variables (n = 474)

***Variable***	***1***	***2***	***3***	***4***	***5***	***6***	***7***	***8***	***9***	***10***	***11***	***12***	***Mean***	***SD***	***Alpha***
1) P self-esteem	1												55.47	6.10	.80
2) T self-esteem	.18***	1											57.28	8.48	.92
3) P self-efficacy	.64***	.17***	1										28.92	3.90	.78
4) T self-efficacy	.16***	.86***	.21***	1									30.01	5.29	.93
5) P self-concept^a^	.91***	.19***	.90***	.20***	1								0.00	0.91	.86
6) T self-concept^a^	.18***	.96***	.20***	.96***	.21***	1							0.00	0.96	.95
7) P self-control	.45***	.13**	.47***	.15***	.51***	.15***	1						20.67	4.05	.86
8) T self-control	.09#	.64***	.14**	.61***	.12**	.65***	.14**	1					23.75	5.57	.93
9) P child–parent relationship	.52***	.05	.41***	.06	.51***	.06	.49***	.05	1				65.54	6.38	.78
10) T child-teacher relationship	.07	.63***	.11*	.60***	.10*	.64***	.15***	.61***	.06	1			68.19	7.71	.89
11) P cumulative adversity index	-.09	-.16***	-.05	-.19***	-.08	-.18***	-.06	-.13**	-.07	-.10*	1		0.00	3.06	n/a
12) P SDQ difficulties (Time 2)	-.43***	-.17***	-.37***	-.24***	-.44***	-.21***	-.38***	-.20***	-.39***	-.16***	.26***	1	8.24	5.43	.83
13) T SDQ difficulties (Time 2)	-.15***	-.26***	-.18***	-.34***	-.18***	-.31***	-.17***	-.34***	-.14**	-.33***	.13**	.42***	6.94	6.21	.87

*Note.* P= parent-reported; T= teacher-reported.

^a.^A child self-concept composite score for each informant was created by averaging together standardised scores for child self-esteem and child self-efficacy.

* *p* < .05. ** *p* < .01. *** *p* < .001.

#### Child’s mental health difficulties

Parents and teachers completed the Strengths and Difficulties Questionnaire (SDQ) [98], a screening questionnaire designed to assess children’s behaviour and emotions. It consists of 25 items divided between five subscales: Emotional Symptoms; Conduct Problems; Hyperactivity; Peer Problems; and Prosocial Behaviour. Respondents provide answers on the basis of the child’s behaviour (e.g., “generally disobedient”) over the previous six months or the current school year, using a three-point response format of “not true” to “certainly true”. Scores on each subscale can range from 0 to 10. An overall emotional-behavioural difficulties score is generated by summing the subscale scores, with the exception of the Prosocial subscale. Scores on Total Difficulties can range from 0 to 40, with higher scores indicating greater mental health difficulties. Total difficulties scores above 14 on parent-reports and above 12 on teacher-reports are considered ‘of concern’ in the abnormal/clinical range. The SDQ has well-established psychometric properties, including strong relationships with diagnostic interviews [99-101].

#### Child’s internal strengths

##### Behavioural self-efficacy

The child’s level of self-efficacy as perceived by the parent and teacher was measured with the Self-Efficacy Scale-Teacher Version [102]. This scale consists of 9 items reflecting self-efficacious behaviours. Items (e.g., “when presented with a new task, the child believes he or she can do it”) are rated with a four-point Likert scale ranging from “not at all like the child” to “like the child”. Items are summed to create a total score ranging from 9 to 36, with higher scores representing higher self-efficacy. The scale has good reliability and factorial validity, and exhibits expected correlations with Conners’ Teacher Rating Scale [102].

##### Behavioural self-esteem

The child’s behavioural self-esteem was measured with the 14-item Behavior Rating Form – Revised [103], which measures young children’s self-esteem as inferred or perceived by a parent or teacher. Each item (e.g., “this child refers to himself/herself in generally negative terms”) is rated using a 5-point Likert scale from “never” to “always”. Total scores are derived by summing items and can range from 14 to 70, with higher scores indicating higher levels of inferred self-esteem. This measure has been found to have high internal consistency [103].

##### Emotional self-control

Parents and teachers completed the self-control subscale of the Devereux Early Childhood Assessment (DECA) [38]. The DECA is a standardised, norm-referenced behaviour rating scale for children aged 2 to 5 years. The 8-item self-control subscale measures children’s ability to experience a range of feelings and express them using appropriate actions and words. Respondents rate the frequency of behaviours exhibited by the child over the last four weeks (e.g., “calm himself/herself down when upset”) on a five-point Likert scale ranging from “never” to “very frequently”. Items are summed so that higher scores indicate greater emotional self-control. The DECA has strong psychometric properties, demonstrating both high internal consistency and reliability, and discriminant validity [38,104,105].

#### Child’s external relationship context

##### Quality of Child’s relationships with parents and teachers

Parents and preschool teachers described the quality of their relationships with the children using the short forms [106] of the Child–Parent Relationship Scale (CPRS) [107] and the Student-Teacher Relationship Scale (STRS) [108], respectively. The two questionnaires contain 15 identical items on which parents and teachers rate their perceptions of their relationship with the child using a 5-point Likert scale (“definitely does not apply” to “definitely applies”). Items assess the level of closeness, warmth, and conflict in the relationship, and are based on behaviours relevant to attachment theory (e.g., “If upset, this child will seek comfort from me”). Total scores are created by summing the 15 items, with higher scores indicating better quality relationships. Both measures have good psychometric properties, including moderate correlations with behavioural ratings of adult-child interaction [108-110].

#### Child’s exposure to familial adversity

Parent-reports at the baseline assessment were used to measure 11 family adversities within five key groups that are consistently associated with higher rates of mental health difficulties among children.

##### Family socio-economic status

Family Socio-Economic Status (SES) was measured with five parent-reported variables. The first two variables were mothers’ and fathers’ level of completed educational qualifications. Third was the family’s paid employment status. To provide a clearer reflection of economic adversity, the employment status of both the mother and father (where present) were combined to reflect the level of full-time-equivalent employment within the family, ranging from both parents being employed full-time through to both parents being unemployed (or where a single parent-family, that parent was unemployed). Fourth was family receipt of any means-tested government welfare benefits for lower-income families. The fifth variable was an indicator of potential economic strain and overcrowding: the number of dependent children living with the study child was dichotomised to indicate if the child lived with three or more siblings [66].

##### Parental separation

Parents reported the child’s past and present living arrangements. Responses on which parental figures were currently living with the child were dichotomised to reflect a single-parent versus two-parent family. Parents also reported whether the child had always lived with two natural parents or not, and if not, the length of time the child had lived with their mother alone, their father alone, or neither natural parent. This information allowed us to calculate the length of time each child had spent during their lifetime living *without* their two natural parents. Scores could range from 0 “none, always lived with two parents” through to 6 “more than five years”, with higher scores indicating a longer period of parental separation.

##### Early parenthood

Parents reported the age of the child’s mother and father. Children’s current age was subtracted from each parent’s age to calculate the mother’s and father’s age when the child was born. Each parent was then categorised as either an adolescent parent (defined as ≤ 20 years at the time of the child’s birth), or not (≥ 21 years) [10].

##### Parental psychological distress

Parental psychological distress and impairment was assessed using the 12-item version of the General Health Questionnaire (GHQ-12) [111]. The GHQ-12 is a widely used screening instrument designed to detect psychological problems in the general population. Respondents indicate their state of general health over the last four weeks relative to their usual state. For example, the respondent is asked whether they have lost much sleep over worry over the last four weeks. There are four possible responses ranging from ‘not at all’ to ‘much more than usual’ (specific responses vary depending on the item). In the present study, the standard binary scoring method was used [112], for which items are scored as 0-0-1-1. Total scores can range from 0 to 12, with higher scores indicating greater parental psychological distress, and a total score of 1 or more classified as indicating a clinical level of psychological distress [113]. The GHQ-12 has well-established psychometric properties, including high sensitivity and specificity in detecting psychiatric cases [112,113].

##### Stressful life events

Stressful life events occurring within the child’s family were assessed using a modified version of the List of Threatening Experiences Questionnaire (LTE-Q) [114,115] that was utilised in the Longitudinal Study of Australian Children [116]. The LTE-Q asks respondents to report experiencing 12 categories of common negative life events involving moderate or marked long-term threat, such as the death of a family member or friend, or a major financial crisis. The current study slightly adapted the wording of the LTE-Q to identify events occurring within the child’s family unit rather than for an individual. Wording changes were based on the Family Inventory of Life Events questionnaire [117,118]. For example, one item was changed from “*you* were seeking work unsuccessfully for more than one month” to “*a parent* was seeking work unsuccessfully for more than one month”. Parents indicated whether or not each life event had occurred in the family over the past 12 months, which was then tallied to create a total score ranging from 0 to 12, with higher scores indicating a greater number of stressful life events experienced within the family. The LTE-Q has demonstrated good reliability and high sensitivity and specificity to independently rated adversity [114].

##### Composite cumulative family adversities index

Scores on these 11 family adversities were combined to create a cumulative family adversities index. First, a multiple regression containing all 11 adversity variables indicated that there was no evidence of multicollinearity within these adversities, so all 11 adversities were included in the cumulative adversity composite score. Bivariate correlations between each adversity variable and SDQ outcomes indicated that the effect sizes were all in the small to trivial range. Given that there was no large variation in effect sizes for the adversities, it was deemed reasonable for the composite adversity score to assume equal weights for each of the 11 adversity factors. This also meant that the composite adversity score used was identical for examining parent-reported and teacher-reported SDQ outcomes, respectively. To ensure that each of the five groups of family adversities (SES, parental separation, early parenthood, parental psychological distress, and stressful life events) was equally weighed in the composite index derived, a standardised average score was computed for the groups of family adversities that had more than one indicator. Then the scores for these five groups of family adversities were standardised and summed to create a total composite family adversity index score (see [52,56,119,120]).

### Procedure

Data collection for the baseline assessment was coordinated by each Preschool Director, with assistance from the research team. At the first assessment, preschool teachers gave consenting parents questionnaires, which were returned to the preschools in sealed envelopes once completed. Teachers completed questionnaires only after parent consent was received. At the second assessment, parents were mailed questionnaires, and returned them to researchers in stamped self-addressed envelopes. Distribution and collection of teacher surveys was facilitated by a nominated liaison person at each school ^b^. At both assessments, parent questionnaires took approximately 30 minutes to complete, and teacher questionnaires took approximately 10 minutes to complete per child. Teachers who were allocated newly commencing students were instructed to wait five weeks before completing questionnaires about these children, in order to allow time to get to know the child. Thus teachers had interacted with children for a minimum of 5 weeks before their ratings about the child were provided. The average number of months children had been at preschool interacting with the teacher was 8.08 months (*SD =* 3.54). The study methodology was approved by the Research Ethics Committees at the Women’s and Children’s Hospital Adelaide, and the South Australian Department of Education and Children’s Services.

### Statistical analyses

We employed four different statistical methodological approaches to operationally define mental health resilience in the context of cumulative family adversity, and analyse correlates thereof. The same hypothesised set of child, family, and preschool resource factors for resilient outcomes was examined within each approach, to enable direct comparison of results between methods. Effect sizes were interpreted based on standard recommendations [121-123]. Each set of analyses utilised the composite preschool family adversity index to represent family adversity. In multivariate analyses, child gender was treated as a covariate. For each different resilience methodology, we examined outcomes at age 6 on: (1) parent-reported child mental health difficulties, and (2) teacher-reported child mental health difficulties. The use of two separate informants to describe children’s behaviour is important because it allows for the consideration of the context within which the behaviour is observed (i.e., home and school) or the different attributions and perspectives held by the informants, and it can provide a test of convergent validity for hypotheses. Discrepancies among informants regarding child psychopathology have been widely documented, and in the absence of a gold-standard assessment, one cannot determine if parents or teachers are more accurate reporters [100,124]. As these informants both provide distinct information about children’s problems in different contexts, their reports are not interchangeable. Researchers who have tested several strategies of combining informant reports ultimately recommend keeping the data as separate, as the unique pattern of context-dependent features becomes lost, and questions regarding context cannot be addressed [124].

The first set of analyses followed the ‘statistical interaction’ approach. Specifically, hierarchical multiple regression was used to determine whether any of the proposed resource factors moderated the relationship between children’s cumulative adversity exposure scores and their mental health difficulties at age 6. A significant interaction effect whereby higher resource levels reduced the effect of adversity on mental health difficulties would indicate a protective effect. Covariates were entered at Step 1. The cumulative family adversity index was entered at Step 2, and all resource factors were entered simultaneously at Step 3. The interaction terms between family adversity and each resource factor were then entered at Step 4. All continuous variables were centred prior to computing interaction terms, and any significant interaction terms were then explored further within plots using Aiken and West’s [125] methods.

The second set of analyses used the ‘residuals’ approach. Linear regression was first used to compute a ‘resilience residuals’ variable, which subsequently became the outcome variable in a second regression with all resource factors entered as predictor variables. In the first regression, the standardised residual ‘resilience’ scores (predicted-obtained discrepancies) were generated by regressing children’s level of mental health difficulties on their cumulative family adversity score. In the present study, in linear regressions the composite family adversity index accounted for 7.0% (*R*^*2*^ = 0.070, *p* < .001) and 1.7% (*R*^*2*^ = 0.017, *p* < .01) of the variance in children’s Time 2 parent-reported and teacher-reported SDQ difficulties scores, respectively, with greater family adversity associated with greater SDQ difficulties. The standardised residual scores generated from these two regressions were then reverse-coded so that higher scores indicated greater mental health resilience on a continuum from vulnerability through to resilience [87]. As a result, children with positive residual scores (i.e., falling above the regression line fitted) showed ‘better than expected’ mental health than predicted by their exposure to adversity, and were considered resilient, to at least some degree. Conversely, children whose mental health was ‘worse than expected’ (i.e., a negative residual) were considered more vulnerable. The size of the residual (i.e., the distance from the regression line fitted) provided an indication of their level of resilience or vulnerability. These residual scores signify the variance in adjustment that is *not* explained by adversity, therefore representing the ‘unexplained variance’ inherent within resilient outcomes [24]. In the subsequent regressions, these resilience residual scores were treated as the outcome variable and were regressed on the covariates, all entered at Step 1, and the proposed resource factors, all entered at Step 2.

The third set of analyses also utilised these resilience residual scores within multiple-group analyses, which were conducted to determine whether the effect sizes of the resource factors on children’s mental health resilience residuals differed between children who were exposed to low- versus high levels of cumulative family adversity.

The fourth set of analyses used the ‘person-centred’ approach. Initially, four key groups of children were identified through a two-step process. First, scores on adversity and the two mental health difficulties variables were divided into thirds, as used in other person-centred resilience investigations (e.g., see [126]). Low and high adversity were defined as the bottom and top thirds on the cumulative adversity variable, and poor and good adjustment were defined as the top and bottom thirds of each SDQ mental health difficulties variable (as this was negatively scored). This tertile approach balances the dual needs of retaining an adequate sample size for each group, and ensuring the ‘low’ and ‘high’ groups are conceptually distinct (which arguably cannot be achieved with a median split). Next, comparisons of groups showing extreme functioning were planned, to determine if resource variables showed different effects in low- versus high-adversity conditions [12,90]. For this reason, children in the middle third on either or both variables were not retained in further analyses ^c^. Thus for the fourth set of analyses, the sample was reduced to 237 children for parent-reported outcomes and 234 children for teacher-reported outcomes. The resulting four groups were labelled ‘Resilient’, ‘Maladaptive’, ‘Competent’, and ‘Highly Vulnerable’, using Masten and colleagues [12] taxonomy, and were then compared on their levels of resources using MANOVA. When the assumption of homogeneity of variances was violated, the non-parametric Kruskal-Wallis (for between-groups effects) and Mann–Whitney Test (for planned comparisons) were used to analyse group differences for those resource variables.

## Results

### Preliminary analyses

Table 1 displays the family adversity background characteristics of participating children. One in 8 children were living in a single parent family, but typically children had spent less than a year living separate from one (or both) of their natural parents in their home. Families had experienced an average of one stressful life event in the past year. A total of 39.1% primary caregiving parents scored above the clinical cut-off on GHQ psychological distress, which is somewhat higher than the percentage found in national surveys of Australian adults [113]. Approximately a quarter of mothers and a third of fathers had not completed high school. However, most families (84.2%) were supported on at least one full-time equivalent job. Nonetheless, 40% of families were receiving a means-tested government welfare benefit. Overall, the sample did not differ appreciably on demographic characteristics from other children in the general Australian population, with similar rates of welfare receipt, employment, and single-parent families [97]. All children had experienced at least some degree of adversity (i.e., no child obtained the lowest possible score on all 11 adversity variables), which is important given that by definition, the presence of adversity is required to be able to exhibit resilience (e.g., [16]). Scores on the composite family adversity index variable ranged from −3.67 to +14.44 (*M* = 0.00, *SD* = 3.06).

Means and standard deviations for continuous variables are also shown in Table 2. Children’s mean scores tended towards the moderate to upper range, suggesting generally healthy child strengths and relationships. Children’s mean SDQ total difficulties scores were within the normal range [98]. At Time 2 (age 6), a total of 8.2% and 10.1% of children scored above the clinical cut-off on the parent-reported and teacher-reported SDQ total difficulties, respectively. This compares to 5.3% and 5.9% for parent- and teacher-reported SDQ scores at Time 1 (age 4). These proportions are fairly similar to those found in national cohorts of young Australian children [127].

We examined the potential role of child gender, child age, and at Time 2, the number of terms at school, school year level, and school type (government or private), as covariates. Only gender was significantly associated with Time 2 SDQ total difficulties (*r* = −.26, *p* < .001 for teacher-reported outcomes; not significant for parent-reported outcomes). Therefore child gender was treated as a covariate in subsequent multivariate analyses.

Table 2 shows the bivariate correlations between the continuous variables, and the means, standard deviations, and Cronbach’s alphas for these variables. As expected, many of the resource variables showed significant moderate positive intercorrelations. Possible multicollinearity was evident between self-esteem and self-efficacy, which were correlated at .64 when reported by parents, and at .86 when reported by teachers. Collinearity diagnostics within multiple regression showed the presence of multicollinearity, particularly for teacher-reported self-esteem and self-efficacy [128]. Thus in this sample it was difficult to distinguish between these two constructs. From a theoretical perspective these constructs originate within a global higher-order self-concept construct, and thus are expected to be highly conceptually interrelated [129]. Thus teacher-reported self-esteem and self-efficacy were combined by averaging their standardised scores to create a composite variable, and the same approach was used for parent-reported self-esteem and self-efficacy. These composite self-concept variables were utilised in subsequent analyses. These parent- and teacher-reported composite self-concept variables had means of zero and *SDs* of 0.91 and 0.96, respectively. Their bivariate correlations with the study variables are shown in Table 2. Also shown in Table 2, the bivariate correlations between parent-reported and teacher-reported scores on child self-concept, child self-control, and SDQ total difficulties respectively were each small but significant and positive. Parent and teacher reports on the SDQ total difficulties score were correlated at .42. While the size of this correlation suggests they may be measuring a similar underlying construct, the informant discrepancy indicates that the children’s behaviour reported at home and at school may be context or informant specific [100,124].

### Approach I: Statistical Interaction Resilience Methodology

#### Bivariate correlates of Children’s mental health difficulties

The bottom of Table 2 displays the bivariate associations between the Time 1 cumulative adversity index and proposed resources, and children’s mental health difficulties as reported by parents and teachers two years later. Higher levels of cumulative family adversity were positively associated with higher child SDQ mental health difficulties. Both parent- and teacher-reported child self-concept and child self-control held significant moderate [121] negative correlations with both parent-reported and teacher-reported child SDQ mental health difficulties. Child–parent relationship quality and child-teacher relationship quality were each significantly negatively associated with both parent-reported and teacher-reported child SDQ mental health difficulties. Whilst all bivariate correlations were significant and in the expected direction, an informant effect was notable – the size of the correlations was considerably larger when the informant was the same for both the predictor and outcome variable.

#### Multivariate correlates of Children’s mental health difficulties

Two hierarchical multiple regressions were conducted assessing Time 1 correlates of parent- and teacher-reported SDQ mental health difficulties at Time 2, respectively. In these regressions, child gender was entered as a covariate at Step 1, followed by the cumulative family adversity index score at Step 2, the main effects of the set of proposed resource variables at Step 3, and finally the interaction terms between family adversity and each of the proposed resource variables at Step 4. ^d^

Results for the regression model predicting parent-reported child mental health SDQ difficulties are shown in Table 3. Greater exposure to cumulative family adversity at Time 1 was associated with greater parent-reported SDQ difficulties two years later. At Step 3, all four resource factors held significant main effects as correlates of subsequent parent-reported child SDQ difficulties: greater child self-concept and self-control, and better quality relationships with parents and preschool teachers, were each associated with fewer SDQ difficulties. The main effects model at Step 3 accounted for 30.4% of the variance in parent-reported SDQ difficulties scores. The addition of the four interaction terms at Step 4 of the model only increased the variance explained by 0.7%. The interaction between family adversity and child-teacher relationship quality approached statistical significance (*p* = .06) in the parent-reported SDQ difficulties model. Because this interaction term did reach statistical significance in post-hoc analyses when child-teacher relationship was assessed separately from the other resource variables (*β* = .09, *p* < .05), we examined the interaction plot (not shown here), which indicated that while the size of this moderated effect was very small, it suggested a “protective-reactive” effect [80,130]; that is, having better child-teacher relationships conferred advantages for better mental health outcomes, but less so under conditions of high family adversity.

**Table 3:** Multiple regressions predicting time 2 child SDQ mental health difficulties (n=474)

	***Parent-reported child mental health SDQ difficulties***			***Teacher-reported child mental health SDQ difficulties***		
***Time 1 predictor variables***	***β***	***R***^***2***^***(ΔR***^***2***^***)***	***ΔF***	***β***	***R***^***2***^***(ΔR***^***2***^***)***	***ΔF***
*Step 1:*		.003 (.003)	1.32		.065 (.065)	32.79***
Gender (female)	-.05			-.26***		
*Step 2:*		.073 (.070)	35.77***		.083 (.018)	9.16***
Gender (female)	-.06			-.26***		
Cumulative Adversity Index	.27***			.13**		
*Step 3:*		.304 (.230)	38.61***		.197 (.114)	16.61***
Gender (female)	-.02			-.19***		
Cumulative Adversity Index	.22***			.08*		
Self-concept^a^	-.26***			-.03		
Self-control^a^	-.14**			-.18**		
Child–parent Relationship Quality	-.17***			-.12**		
Child-Teacher Relationship Quality	-.08*			-.15**		
*Step 4:*		.311 (.007)	1.22		.209 (.012)	1.80
Gender (female)	-.01			-.19***		
Cumulative Adversity Index	.23***			.07		
Self-concept^a^	-.26***			-.02		
Self-control^a^	-.15**			-.18**		
Child–parent Relationship Quality	-.16***			-.11**		
Child-Teacher Relationship Quality	-.09*			-.16**		
Adversity x self-concept	.04			-.06		
Adversity x self-control	-.02			-.08		
Adversity x child–parent relationship	-.03			.05		
Adversity x child-teacher relationship	.08#			.06		

*Note.*^a^ Models predicting parent-reported child mental health difficulties included parent-reported self-concept and self-control as predictor variables, and models predicting teacher-reported child mental health difficulties included teacher-reported self-concept and self-control as predictor variables.

# *p* < .10; * *p* < .05; ** *p* < .01; *** *p* < .001.

The regression model predicting teacher-reported child SDQ difficulties is also shown in Table 3. Regarding main effects, it can be seen that girls had significantly lower teacher-reported child SDQ difficulties than boys. Greater exposure to cumulative family adversity at Time 1 was associated with greater teacher-reported child SDQ difficulties two years later (Step 2 and 3). The addition of the four proposed resource factors at Step 3 indicated that greater child self-control, better quality child–parent relationships, and better quality child-teacher relationships were each associated with lower teacher-reported SDQ difficulties two years later. There was no significant main effect for self-concept. The main effects model at Step 3 accounted for 19.7% of the variance in teacher-reported SDQ difficulties scores. The addition of the four interaction terms at Step 4 of the model increased the variance explained by only 1.2%, and none of these interaction terms were significantly associated with teacher-reported SDQ difficulties. ^e^

Overall, the findings from these interaction model analyses suggest that the resource factors examined tended to have a more general promotive capacity for all children regardless of their level of exposure to adversity, rather than a specific protective effect present only for children who have faced significant adversity.

### Approach II: Residuals Resilience Methodology

Scores on parent-reported child mental health resilience generated from the regression residuals ranged from −4.28 to +2.31, and from −3.67 to +1.64 for teacher-reported child mental health resilience. By definition, the standardised residuals had mean scores of zero and *SD*s of 1. On parent-reported SDQ, 57% of children had better outcomes than expected (i.e., a positive residual) and thus were considered to be exhibiting at least some degree of mental health resilience. For teacher-reported SDQ, this percentage was 60.5%. Scores on parent- and teacher-reported child mental health resilience showed a significant moderate positive correlation (*r* = .40, *p* < .001).

#### Bivariate correlates of Children’s mental health resilience residuals

Table 4 shows the bivariate correlations among both measures of mental health resilience residuals, and the set of proposed resources for the whole sample (*n*= 474). Both parent- and teacher-reported child self-concept and self-control held significant moderate positive correlations with both parent- and teacher-reported mental health resilience residuals. Child–parent relationship quality and child-teacher relationship quality were each significantly positively associated with both parent- and teacher-reported child mental health resilience residuals. Again, an informant effect was notable, with correlations larger when the informant was the same for both variables.

**Table 4:** Bivariate correlations between resource factors and child mental health resilience residuals – for the whole sample, and for low and high adversity groups

	***Parent-reported child mental health resilience residuals***			***Teacher-reported child mental health resilience residuals***		
***Time 1 predictor variables***	***Whole sample r (n=474)***	***Low adversity r (n=158)***	***High adversity r (n=159)***	***Whole sample r (n=474)***	***Low adversity r (n=158)***	***High adversity r (n=159)***
Gender (female)	.06	.06	.01	.26***	.26***	.25**
P self-concept	.44***	.45***	.42***	.17***	.23**	.19*
T self-concept	.17***	.21*	.19*	.29***	.28***	.38***
P self-control	.38***	.39***	.37***	.17***	.13#	.17*
T self-control	.17***	.25**	.15#	.33***	.29***	.38***
P Child–parent relationship quality	.38***	.43***	.41***	.13**	.19*	.08
T Child-teacher relationship quality	.14**	.31***	.01	.32***	.36***	.26***

*Note.* P = parent-reported variable; T = teacher-reported variable.

# *p* < .10; * *p* < .05; ** *p* < .01; *** *p* < .001.

#### Multivariate correlates of Children’s mental health resilience residuals

Two separate hierarchical multiple regressions were conducted assessing Time 1 correlates of each of the two measures of mental health resilience residuals: that reported by parents and that reported by teachers. The two-step multiple regression model predicting parent-reported child mental health resilience residuals is shown in Table 5 under the sub-heading ‘whole sample’. The final model including all 4 predictor variables and the covariate gender accounted for 24.9% of the variance in children’s parent-reported mental health resilience residuals at age 6. Standardised regression coefficients (*β*) for each predictor variable in the final model are shown in Table 5. Higher parent-reported child self-concept and self-control, higher quality child–parent relationship, and higher quality child-teacher relationship were significantly associated with greater parent-reported child mental health resilience residuals two years later.

**Table 5:** Multiple regressions predicting child mental health resilience residuals – for the whole sample, and for low and high adversity groups

	***Parent-reported mental health resilience residuals***			***Teacher-reported mental health resilience residuals***		
***Time 1 predictor variables***	***Whole sample***	***Low adversity***	***High adversity***	***Whole sample***	***Low adversity***	***High adversity***
	***β (n = 474)***	***β (n = 158)***	***β (n = 159)***	***β (n = 474)***	***β (n = 158)***	***β (n = 159)***
*Step 2:*						
Gender (female)	.02	-.11	.08	.19***	.17*	.21**
Self-concept	.27***	.27***	.22*	.02	.04	.19^#^
Self-control	.14**	.14^#^	.16^#^	.18**	.07	.24*
Child–parent relationship quality	.17***	.22**	.25**	.12**	.13^#^	.08
Child-teacher relationship quality	.08^*^	.25***	-.04	.15**	.24*	-.01
*R*^*2*^	.25	.34	.26	.18	.19	.21
*F*	31.00***	15.43***	10.47***	20.71***	7.14***	8.35***
*ΔR*^*2*^	.25	.34	.26	.11	.12	.15
*ΔF*	38.20***	19.12***	13.08***	16.28***	5.79***	7.41***

*Note.* Models predicting parent-reported child mental health resilience residuals included parent-reported self-concept and self-control as predictor variables, and models predicting teacher-reported child mental health resilience residuals included teacher-reported self-concept and self-control as predictor variables. Variable coefficients are standardised regression coefficients (Betas). Step 1 of the model adjusted for the covariate child gender, with the resource variables entered at Step 2.

# *p* < .10; * *p* < .05; ** *p* < .01; *** *p* < .001.

The model predicting teacher-reported child mental health resilience residuals is also shown for the whole sample in Table 5, with the final model accounting for 18.1% of the variance in scores. Three Time 1 predictor variables were significantly associated with Time 2 teacher-reported child mental health resilience residuals, in addition to gender. Higher quality relationship between the child and their parent and the child and their preschool teacher, and higher teacher-reported child self-control were significantly associated with greater teacher-reported child mental health resilience residuals. ^f^

### Approach III: Multiple-Groups Residuals Resilience Methodology

The above bivariate correlations and multiple regressions predicting resilience residuals were then run separately for children facing low-adversity (with scores in the bottom third on the family adversity composite index) and high-adversity (with scores in the top third) ^g^ , allowing the effect sizes for each group to be compared. The bivariate correlations for these two sub-groups are shown in Table 4. The effect sizes for each resource variable were generally small to moderate, and were in most cases very similar across the groups. The largest difference between the low-adversity and high-adversity groups were seen on child-teacher relationship quality – for parent-reported resilience residuals, the effect of positive child-teacher relationship was .30 larger for children with low-adversity than for those facing high-adversity, indicating a small “reactive” effect (i.e., conferring greater benefit for children with low-adversity).

The multiple regression results for the low-adversity and high-adversity groups are displayed in Table 5. For the model predicting parent-reported mental health resilience residual scores, there were only small differences in the effects of resource variables at low and high levels of adversity. Among children experiencing low-adversity, significant Time 1 correlates of parent-reported resilience residuals were self-concept, child–parent relationship quality, and child-teacher relationship quality, all showing small positive effects [121]. However, significant Time 1 correlates among children facing high-adversity were self-concept and child–parent relationship quality (with small positive effects). For child-teacher relationship quality, effect sizes were smaller among children facing high-adversity, with a beta difference between the adversity groups of .29. Whilst this difference was small, it resembled a “reactive” effect, conferring higher benefits among the group exposed to low-adversity: child-teacher relationship quality did not show any benefits for children experiencing high-adversity. There was little difference in betas between low- and high-adversity groups on self-concept, self-control, and child–parent relationship quality, indicating they could be considered to have small but generally promotive effects.

For the model predicting teacher-reported mental health resilience residual scores, there was some small differences in the effects of resource variables between the low- and high-adversity groups. Self-concept and child–parent relationship did not reach significance as correlates of resilience residuals in low- or high-adversity groups. Among children facing low-adversity (but not those facing high-adversity), child-teacher relationship quality was significantly associated with teacher-reported resilience residuals, showing a small positive effect. Child-teacher relationship quality resembled a small “reactive” effect (conferring greater benefit among low-adversity children), with a between-group beta difference of .25. Among children facing high-adversity (but not those facing low-adversity), self-control was significantly associated with resilience residuals, showing a small positive effect. This beta difference of .17 resembled a small “protective” effect, with greater self-control conferring greater protection under conditions of high-adversity.

### Approach IV: Person-Centred Resilience Methodology

#### Formation of adaptation groups

For both parent-reported and teacher-reported outcomes, children were classified into four key groups based on their combinations of scores on the adversity (lowest/highest tertile) and mental health difficulties (lowest/highest tertile) variables. When using parent-reported mental health difficulties, this classification yielded 39 Resilient (high adversity, low mental health difficulties), 79 Maladaptive (high adversity, high mental health difficulties), 72 Competent (low adversity, low mental health difficulties), and 47 Highly Vulnerable (low adversity, high mental health difficulties) children. When using teacher-reported mental health difficulties, classification yielded 50 Resilient, 71 Maladaptive, 65 Competent, and 48 Highly Vulnerable children. Chi-square tests for independence indicated that the mental health difficulties were not evenly distributed across the adversity groups, when using both parent-reported mental health difficulties *χ*^*2*^ (4) = 18.63 (*p* < .001), and teacher-reported mental health difficulties, *χ*^*2*^ (4) = 7.40 (*p* = .10). For instance, 46% of the children scoring in the lowest adversity group showed low parent-reported mental health difficulties (the ‘Competent’ children), whereas only 25% of children scoring in the highest adversity group showed low parent-reported child mental health difficulties (the ‘Resilient’ children), *z* = 4.02, *p* < .001. Although less apparent, this effect was present when using teacher-reported mental health difficulties (41% of low-adversity children showed low mental health difficulties, compared with 31% of high-adversity children, *z* = 1.88, *p* = .06). Thus, consistent with the definition of resilience as ‘unexpected’ positive adaptation, children experiencing high levels of adversity were significantly less likely to show low levels of mental health difficulties compared with their low-adversity counterparts.

Additionally, MANOVAs were conducted on adversity and mental health difficulties scores to ensure the creation of groups had worked as intended. As a direct result of the cut-off method, the Resilient and Competent children did not differ on their levels of mental health difficulties, and the Resilient and Maladaptive children did not differ on their levels of adversity.

#### Comparison of groups

Next, MANCOVAs were conducted to determine whether the resource variables were associated with parent-reported and teacher-reported adaptation group classification. All resource variables held good internal consistencies when examined for each group separately (Cronbach’s Alphas from .75 to .96). Within MANCOVA (after adjusting for child gender), there was a statistically significant difference on the combined resource variables between (i) the four ‘parent-reported’ adaptation groups (*Wilks’ λ* = .69, *F* (15, 632) = 6.05, *p* < .001), and (ii) the four ‘teacher-reported’ adaptation groups (*Wilks’ λ* = .76, *F* (15, 625) = 4.45, *p* < .001). Both demonstrated a medium effect size (*partial η*^*2*^’s of .12 and .09, respectively).

Mean scores for the parent-reported and teacher-reported adaptation groups on each resource variable are displayed in Table 6. The *F* values in Table 6 represent the univariate between-subjects tests for each resource variable, which were adjusted using the Bonferroni procedure. The effect of the covariate gender was also partialed-out of these results (not shown in Table 6 for ease of presentation). Results indicated that ‘parent-reported’ adaptation group was significantly associated with parent-reported self-concept, self-control, child–parent relationship quality, and child-teacher relationship quality (see *F* values in Table 6). With the exception of child-teacher relationship quality, these resource variables showed large effect sizes (see *partial η*^*2*^ values in Table 6). Also shown in Table 6, ‘teacher-reported’ adaptation group was significantly associated with teacher-reported self-concept, self-control, and child–parent relationship quality, with small to medium effect sizes (see *F* and *partial η*^*2*^ values in Table 6). Child-teacher relationship quality was not significantly related to ‘teacher-reported’ adaptation group.

**Table 6:** MANCOVA results: resource variable means (and SDs) for the four adaptation groups, between-subjects effects, and planned contrasts

***Resource variable***	***1. Resilient (high adv+ low SDQ)***	***2. Maladaptive (high adv+ high SDQ)***	***3. Competent (low adv+ low SDQ)***	***4. Highly-vulnerable (low adv+ high SDQ)***	***Between-subjects effects***		***Planned contrasts***
					***F***	***Partial η***^***2***^	
Parent-reported adaptation groups (*n* = 237)							
	(*n* = 39)	(*n* = 79)	(*n* = 72)	(*n* = 47)	*df* = 3, 233		
P Child–parent relationship^a^	68.23 (5.80)	62.70 (6.61)	68.84 (4.67)	63.40 (6.37)	45.42***	.19	1 > 2; 1 = 3; 3 > 4
T Child-teacher relationship^a^	68.36 (7.14)	66.53 (8.64)	70.03 (5.51)	66.38 (9.41)	7.48*	.04	1 = 2; 1 = 3; 3 = 4
P Self-concept	0.39 (0.76)	−0.46 (0.94)	0.44 (0.71)	−0.39 (0.83)	21.31***	.22	1 > 2; 1 = 3; 3 > 4
P Self-control	22.23 (4.08)	19.09 (4.15)	22.19 (3.52)	19.13 (4.37)	11.84***	.13	1 > 2; 1 = 3; 3 > 4
Teacher-reported adaptation groups (*n* = 234)							
	(*n* = 50)	(*n* = 71)	(*n* = 65)	(*n* = 48)	*df* = 3, 230		
P Child–parent relationship	65.16 (7.05)	64.44 (7.04)	67.61 (5.55)	64.78 (6.95)	3.04*	.04	1 = 2; 1 = 3; 3 = 4
T Child-teacher relationship^a^	68.10 (5.79)	65.70 (9.80)	69.65 (5.30)	66.13 (10.21)	4.45	.04	n/a
T Self-concept^a^	−0.01 (0.87)	−0.60 (1.09)	0.27 (0.66)	−0.07 (1.07)	23.51***	.12	1 > 2; 1 = 3; 3 = 4
T Self-control^a^	24.77 (4.62)	20.90 (5.56)	24.85 (4.71)	22.63 (6.85)	20.43***	.09	1 > 2; 1 = 3; 3 = 4

*Note*. These results adjust for the covariate gender (not shown for ease of presentation). For the three planned contrasts, all significant group differences found were at *p* < .05. Numbers within the planned contrasts column refer to adaptation group shown in the mean scores column headings. P = parent-reported variable; T = teacher-reported variable; adv = adversity; SDQ = total SDQ mental health difficulties score; n/a = planned contrasts not conducted as no significant univariate group differences.

^a^ Non-parametric tests used (Kruskal-Wallis Test for between-subjects, Mann–Whitney Test for paired comparisons) due to unequal variances across groups. In these cases, a *χ2* value is reported instead of an *F* value.

Next, a series of three planned comparisons were made for each of the univariately significant resource variables, to determine if differences existed between (1) the Resilient and the Maladaptive group, (2) the Resilient and the Competent group, and (3) the Competent and Highly Vulnerable group. These are first reported for the ‘parent-reported’ adaptation groups, and then for the ‘teacher-reported’ adaptation groups. Results are presented in Table 6.

##### Parent-reported planned comparisons

For parent–child relationship quality, Resilient children were rated significantly higher than Maladaptive children (large effect size, *d* = .89). Additionally, Competent children showed significantly higher levels than did the Highly Vulnerable children (large effect size, *d* = .97). The Resilient and Competent children did not differ. For self-concept, Resilient children were rated significantly higher than Maladaptive children (large effect size, *d* = .99). Additionally, Competent children showed significantly higher levels than Highly Vulnerable children (large effect size, *d* = 1.07). The Resilient and Competent children did not differ. For self-control, Resilient children were rated significantly higher than Maladaptive children (moderate effect size, *d* = .76), Competent children showed significantly higher levels than Highly Vulnerable children (moderate effect size, *d* = .77), and the Resilient and Competent children did not differ. None of the three planned comparisons were significant for child-teacher relationship quality. The overall pattern of group differences suggested that all three variables functioned as promotive factors; that is, higher levels of child–parent relationship quality, self-concept and self-control were associated with lower levels of mental health difficulties, regardless of the level of adversity experienced.

##### Teacher-reported planned comparisons

For self-control, the Resilient children were rated as significantly higher than Maladaptive children (moderate effect size, *d* = .76). However, there was no significant difference between the Competent children and the Highly Vulnerable children. Furthermore, the Resilient and Competent children did not differ. Altogether, these effects suggested that self-control worked as a protective factor, where higher self-control levels led to lower levels of mental health difficulties, specifically under conditions of high-adversity exposure. For self-concept, the Resilient children were rated as significantly higher than Maladaptive children (moderate effect size, *d* = .60). However, there was no significant difference between the Competent children and the Highly Vulnerable children. Furthermore, the Resilient and Competent children did not differ. Again, this pattern suggested that self-concept worked as a protective factor, where higher self-concept levels led to lower levels of mental health difficulties, specifically under conditions of high-adversity exposure. For child–parent relationship quality, none of the three planned comparisons were significant.

### Sensitivity Analyses: Prospective Longitudinal Antecedents

In order to determine the sensitivity of the resource factors as predictors of the onset of new mental health difficulties in addition to correlates of the subsequent absolute level of mental health difficulties, we replicated the statistical analyses described above for a reduced sample of 425 children for whom there was no evidence of mental health difficulties at the Time 1 baseline assessment. The 49 children who scored above the clinical cut-off on either parent- or teacher-reported SDQ difficulties at baseline were excluded. Following the guidelines of Kraemer and colleagues [131], this sensitivity analysis allowed the examination of whether the proposed resource variables could prospectively predict (as temporal antecedents) the onset and escalation of mental health difficulties between age 4 and age 6, over and above their synchronous association with baseline mental health difficulties.

Smaller effect sizes and lower proportion of variance explained were evident throughout the sensitivity analyses using all four resilience methodologies, possibly suggesting that the associations are mediated through preschool mental health difficulties. The largest difference from previously reported results was that the small effects (both protective and promotive) of child-teacher relationship on parent- and teacher-reported mental health outcomes did not persist when children with pre-existing mental health difficulties were excluded. Furthermore, the small promotive effect of child–parent relationship quality on teacher-reported mental health outcomes present on all resilience methodologies in the former analyses was no longer apparent in the prospective analyses excluding children with clinical-level mental health difficulties at baseline. Nonetheless, several resource factors were prospectively predictive of subsequent mental health outcomes in the context of adversity. Greater child self-concept, self-control, and child–parent relationship quality continued to demonstrate small promotive effects on parent-reported child mental health outcomes in all four resilience methodologies. On teacher-reported mental health outcomes, self-control continued to indicate a small protective effect according to the person-centred and the multiple-groups methodologies and a small promotive effect when examining statistical interactions. Self-concept continued to have little apparent effect on teacher-reported mental health outcomes. Statistical tables showing these results are available upon request from the corresponding author.

## Discussion

This study aimed to identify resource factors during preschool that were associated with early childhood mental health resilience in the context of cumulative family adversity. As is conditional within resilience research, we found a small positive association between cumulative family adversity experienced during preschool and the level of childhood mental health difficulties reported by parents and teachers two years later, consistent with previous research [12,90]. Inherent to the phenomenon of resilient outcomes as ‘unexpected adaptation’, the individual differences in how children responded to adversity exposure was demonstrated by a notable proportion of the variance in their mental health difficulties being left unexplained by family adversity. We found that several resource factors were bivariately associated with these ‘unexpected’ outcomes, such that higher levels of parent–child relationship quality, teacher-child relationship quality, self-concept, and self-control were positively related to resilient outcomes, or ‘better than expected’ mental health outcomes in the context of children’s levels of family adversity. Furthermore, greater child–parent relationship quality, greater self-concept, and greater self-control during preschool were prospectively found to be antecedent predictors of subsequent mental health difficulties within the context of adversity. These variables have consistently been identified as associated with better mental health outcomes for at-risk children (e.g., [17,26,28,37]), although rarely examined during the preschool and early school years. Together, these results indicate that the correlates and antecedents of mental health in the context of family adversity in young Australian children appear concordant with those found in older children and children in other western countries.

While the effect sizes of these resource factors were generally small to moderate, they were fairly robust correlates of resilience, being related to good mental health outcomes in the presence of adversity bivariately, but also uniquely, when all other resource variables were adjusted for (the main exception to this was self-concept in regards to teacher-reported outcomes). Furthermore, our sensitivity analyses indicated that greater child self-concept, self-control, and child–parent relationship quality were prospective antecedents of subsequent parent-reported resilient mental health outcomes. These results suggest that more can be gained within intervention programmes with every additional resource that is promoted. This aligns with the contention that ‘cumulative protection’ is needed to counteract cumulative adversity [5,18]. The results also highlight the importance of promoting factors from several systems, including the family, school, and the child, to achieve the largest benefit [4]. Additionally, in most cases these resources were found to have predominately promotive effects, generally being beneficial for children experiencing both low- and high- levels of adversity. Thus, these resource factors may be well-suited for use in universal prevention strategies, given that promoting high quality child-adult relationships, positive child self-concept and good self-control may benefit the mental health of all children, regardless of whether they have yet experienced significant family adversity.

Consistent with previous research (e.g., [12]), person-centred analyses suggested that the Resilient children were similar to the Competent children (who had lower levels of adversity) on every resource variable. This finding highlights the ‘self-righting tendencies’ within human development [26], where children generally achieve good outcomes if certain resources are available, even in the presence of adversity.

A unique aspect of the present study was the ability to directly compare the results of several different resilience methodologies. It is noteworthy that the four different methodologies utilised led us to fairly similar conclusions. Even though these methods approached the operationalisation of resilience in slightly different ways, there were only some variations in results. First, the resource variables showed similar effect sizes on mental health outcomes within all methods. Second, the notable reduction in the effect of child-teacher relationship on mental health outcomes in the prospective longitudinal analyses predicting new mental health difficulties was apparent within all resilience methodologies used. Third, significant resources tended to show predominately promotive rather than protective (or interactive) effects on the different methodologies. This convergent evidence suggests that results are not necessarily an artefact of the type of analysis used. It also provides validation for our operationalisation of resilience, and for the construct of resilience more broadly.

We did find some evidence of protective effects in our analyses. In variable-centred interactions and multiple-group methods, child-teacher relationship quality showed a very small protective-reactive effect, conferring greater benefits for children facing low-adversity than high-adversity. However this small effect disappeared in sensitivity analyses prospectively predicting the onset of mental health difficulties. The most robust finding regarding protective effects was for teacher-reported self-control, which demonstrated a protective effect on teacher-reported mental health outcomes in the multiple-groups and person-centred methodologies, but not in the potentially lower-powered statistical interaction model. Greater self-control during preschool conferred greater protection to children under conditions of high-adversity. This effect was evident in both the analyses of absolute mental health outcomes at age 6, and the sensitivity analyses prospectively predicting age 6 mental health outcomes in children with no pre-existing mental health difficulties in preschool.

It must be noted that an ‘informant’ effect was present within our findings: although both parent-reported and preschool teacher-reported resource factors were bivariately related to children’s mental health difficulties as reported by either parents or school teachers, the effect sizes were larger when the informant-type was the same, and some of the associations between variables assessed with different informants diminished to non-significance in multivariate analyses. Overall, the strongest effects were detected when assessing associations between parent-reported resources and subsequent mental health reported by the parent. Consequently, the associations found may be partly due to shared method variance for parent-reported mental health outcomes. However, this informant effect may also be a result of children’s self-concept, self-control, and mental health being context specific. Although parent and teacher-reported resources were not strongly associated with each other, it seems they were related to mental health outcomes in a similar manner. In sum, although children’s self-concept and self-control may manifest or be perceived differently at home and at preschool, the manners in which they influence their mental health appear to be similar.

The findings of this study should be interpreted within the context of the following limitations. First, whilst a strength of this study was the inclusion of reports from two informants, our sole reliance on survey methodology poses limitations on the interpretations of our findings. It is possible that the use of direct observations, child interviews, or diagnostic interviews may have changed the pattern of results. These more objective assessment methods were unfortunately beyond the scope of this study. The reliance on parent and teacher informant reports also meant parents and teachers had to infer children’s internal self-beliefs and emotions from behavioural manifestations associated with these internal child constructs [16]. It is unclear how accurately parents’ and teachers’ perceptual judgements regarding these internal characteristics would correspond to children’s own self-assessments (although our ability to obtain accurate information from children themselves is limited by the young age of the sample).

Second, even though this study included analyses that examined longitudinal preschool correlates of absolute levels of mental health outcomes at school, and prospectively antecedent preschool predictors of the onset or escalation of mental health difficulties once at school, our results can still only suggest but not confirm possible causal sequences. Examining the potential reciprocal or transactional processes between child-adult relationships, self-concept, self-control, and mental health outcomes within family adversity was beyond the scope of this study.

Third, a limitation of key pertinence for studies of resilience is that within our community-based sample, few children had experienced very high levels of family adversity. While our cumulative family adversity index showed sufficient variability, and the association between adversities and mental health difficulties was similar in magnitude to those found in other studies (e.g., [12,90]), overall this association was relatively weak, particularly in comparison to those between some resources and mental health. The ability to detect the degree of moderation by resource factors on the association between adversity and mental health may have been compromised by the modest level of cumulative adversity in the sample [66]. Further exacerbating the underrepresentation of children facing higher levels of adversity in this study was the higher rate of attrition for children facing greater adversity. Therefore, our results may be less generalisable to children facing great adversity, and it is unclear whether the resources we identified as promotive would maintain their beneficial effects at extremely high levels of adversity. It is also not known if our findings would generalise to children in other regions, or whether within-preschool clustering effects influenced our results.

Given that only a minority of children experienced both high adversity and low mental health difficulties within person-centred analyses illustrates that although a number of children showed resilient functioning, they were fighting against the odds. This highlights the need for further research to determine how such children manage to transcend their family circumstances, when many others become engulfed by them. It would be worthwhile to determine whether preschool-age relationships, self-concept, and self-control are equally important for different subcategories of mental health difficulties in young children (e.g., internalising and externalising problems). More research on the preschool-age correlates of resilience within other developmentally relevant domains, such as social and academic functioning, would provide a more complete picture of resilience during early childhood. Because the resource factors included in this study did not explain the majority of the variance in mental health, it is clear that other resource variables are involved in the development of mental health resilience. It would be worthwhile to examine the role of relationships with other important adults (e.g., grandparents, regular carers), and the role of other potential internal characteristics (e.g., optimism) in understanding early childhood resilience to mental health difficulties. Furthermore, the role of biological processes is a recently burgeoning field within resilience research, and future investigations would benefit from the consideration of such factors alongside other child, family, and wider social factors previously implicated in the development of resilient outcomes [20,66,120]. Researchers should also conduct prospective longitudinal research in order to investigate the temporal precedence of preschool-age resource factors and subsequent mental health resilience, and whether these resources are able to predict change over time in resilience. Examining the accumulation of family adversities over several time points rather than just one, and the use of weights on each adversity based on the size of their association with mental health, would also provide a more complete and realistic picture of the influence of family adversity on mental health in young children.

There are several other worthwhile avenues for future research. Given that the methodologies we used in combination provided a holistic view of resilience in young children, we urge other researchers to conduct multiple-methodology resilience studies. This will help to determine if any results found are likely to be reflecting real effects, or if they are artefacts of the analysis method. Further evidence of convergent findings across methods will bolster the construct validity of resilience [80]. Alternatively, if other studies do not find such convergence, the pattern of results may provide researchers with important information on differences in resilience measurement techniques, and perhaps eventually lead the field towards the adoption of a consistent methodology. As there are few multiple-methodology resilience studies from which to draw firm conclusions, much more research needs to be conducted in this area.

In the present study we found evidence of child, family, and school resources for mental health resilience, as well as indication that these resources were interrelated. Furthermore, in some cases the effects of particular resources were diminished when several resources were considered together in multivariate analyses. Such findings suggest that future resilience studies should examine the possible mediating processes by which resource factors exert their effects on mental health [81,132]. For example, Luthar and Brown [132] assert that “relationships lie at the roots of resilience … the presence of support, love, and security fosters resilience in part by reinforcing people’s innate strengths” (p.947). The resilience residuals methodology in combination with multiple-groups resilience methodology provides a valuable platform for the examination of meditational pathways and processes at play between the resource factors in their prediction of resilient outcomes. Because resources found to catalyse the development of other resources are most likely to have the greatest benefits within interventions, information generated from such studies may provide valuable insight into which resource factors should be prioritised within intervention strategies [81,132].

## Conclusion

In drawing the findings from this multiple-methodology study together, the many internal child and external environmental resources for child mental health resilience identified in this study reinforces that early intervention strategies developed will need to be multifaceted in order to address the complexities of the development of childhood resilience. Boosting positive child-adult relationships, self-concept and self-control as resources in early childhood may hold promise for helping children establish a firm foundation that will carry them forward into healthy futures, regardless of what adverse family circumstances come their way.

## Endnotes

^a^Preschool is a government-funded programme which is available to all four year-old children in the year immediately prior to commencing formal schooling. In this 12 month period, 11 to 15 hours per week of preschool education is provided free of charge. While attending preschool in South Australia is not compulsory, most children do: approximately 93% of eligible four year olds attended government-funded state preschools in South Australia from 2006–2007 [133].

^b^Children were tracked regardless of their school destination and were attending 92 different schools across Australia. The majority (69%) were attending government schools in the same district that they had attended preschool. Schools with less than 5 participating children were directly sent teacher surveys by mail.

^c^We also conducted analyses including children within the ‘middle’ groups, to enable more direct comparison between resilience methods by using the same sample size. However, results were almost identical to those that did not include the ‘middle’ group. For ease of presentation, only the results for the 4 extreme groups are reported here.

^d^We also tested models including both parent-reported and teacher-reported self-concept and self-control as predictor variables of SDQ difficulties, in addition to the informant-specific models presented here. The addition of both informants on these predictor variables and their interaction terms made little improvement to the variance explained in the models (*R*^*2*^ increases of .005 and .005 for parent-reported and teacher-reported child SDQ difficulties respectively), and made very little change to the size of most *β* coefficients.

^e^In post-hoc analyses for both parent-reported and teacher-reported SDQ difficulties, we also examined each predictor variable separately in a series of hierarchical multiple regressions to determine their total main effects and interactions with adversity. Whilst all of the predictor variables showed significant main effects on both parent-reported and teacher-reported child mental health difficulties, none of the interaction terms were statistically significant, with the exception of the interaction between adversity and child-teacher relationship in its association with parent-reported SDQ difficulties.

^f^We also tested 7-variable models including both parent-reported and teacher-reported self-concept and self-control as predictor variables of mental health resilience residuals, in addition to the 5-variable models presented. The addition of both informants on these predictor variables made little improvement to the variance explained in the models (*R*^*2*^ increases of .006 and .005 for parent-reported and teacher-reported child mental health resilience respectively), and made very little change to the size of most *β* coefficients.

^g^Children classified in the high adversity group (highest tertile on the adversity composite index) had indeed experienced significantly high levels of family adversity, e,g., 85% of this group had experienced 3 or more adversities, compared to 0% in the low adversity group and 8.5% in the moderate adversity group. Of the children in the high adversity group, 88% lived in a family receiving a government benefit, 42% were living in a single-parent family, 51% of mothers and 64% of fathers had not completed high school, 54% had a parent scoring above the clinical cut-off on the GHQ, and 43% had experienced 2 or more stressful life events in the past 12 months.

## Competing interests

The authors declare that they have no competing interests.

## Authors’ contributions

L.M-L. contributed to the conception and design of the study, data collection, data analysis and interpretation, and drafted the manuscript. A.S. assisted in the design, data collection, data analysis and interpretation for the study, and helped draft the manuscript. M.S. participated in the design of the study, interpretation of data, and drafting and revising the manuscript. P.B. participated in the design of the study, provided statistical advice and data interpretation, and critically revised the manuscript. D.H. assisted with data analysis and helped to draft and revise the manuscript. All the authors have read and approved the final manuscript.
